# Enhancing anemia diagnostics and accessibility in India: a policy recommendation for effective anemia management

**DOI:** 10.3389/frhs.2025.1529094

**Published:** 2025-05-15

**Authors:** Shyamkumar Sriram, Soni Sharma

**Affiliations:** Department of Rehabilitation and Health Services, University of North Texas, Denton, TX, United States

**Keywords:** anemia, public health, diagnostic, politics, women, children, public awareness

## Abstract

Anemia remains a major public health issue in India, with a high prevalence among women, children, and vulnerable populations. Current diagnostic methods vary, impacting the accuracy and comparability of data across regions and complicating anemia tracking. Policy proposals include standardizing diagnostic techniques, enhancing non-invasive device accuracy, and expanding access to diagnostic instruments in underprivileged areas. Strengthening anemia data collection and increasing public awareness are essential for effective anemia management.

## 1 Introduction

Anemia, defined by low hemoglobin levels, affects more than half of India's women and children, with serious health consequences such as reduced physical and cognitive growth in children and decreased productivity in adults. Given these consequences, effectively tracking anemia is critical for successful policy initiatives, especially in resource-constrained places where the incidence is highest. The prevalence of anemia in Indians, especially women, has stayed about 45% since 1990 despite increased national and international attention and current government preventative initiatives and it is strongly linked to iron deficiency (1). Several techniques have been used to determine Hb concentration, but variances in measuring methodologies and resources available across locations frequently result in inconsistencies in anemia results. The National Family Health Survey (NFHS)-5 survey highlighted the need to manage anemia, particularly in rural and low-income communities where it is most prevalent. NFHS has historically given critical anemia statistics, but changes in diagnostic procedures and subsequent exclusions from the program have caused concerns about data integrity and precision. Accurate diagnosis and successful treatment strategies are critical for treating anemia. However, challenges remain in obtaining precise and reputable anemia data across India. Diagnostic inequalities exist because of methodology differences (for example, capillary vs. venous blood), sample processing, and laboratory capacity, all of which alter the accuracy of hemoglobin measurement. With a high national occurrence and global targets aimed at a significant reduction, proper anemia care in India is critical. The purpose of this policy brief is to assess current diagnostic alternatives, investigate policy solutions, and make concrete recommendations to improve anemia diagnosis across India.

## 2 Addressing the burden of anemia

### 2.1 Prevalence statistics

Anemia is a significant public health concern worldwide, impacting roughly 1.8 billion people and resulting in 50.3 million years of disability in 2019 (2). In 2023, the actual prevalence was slightly higher, but similar, indicating a slow positive trend towards improvement.Anemia is especially prevalent in India, with the National Family Health Survey-5 (NFHS-5) finding that 57% of women aged 15%–49% and 67% of children under five are anemic (3). The forecasted anemia prevalence among women aged 15–49 in India is expected to drop from 2024 to 2028 from 52.8 percent in 2024 to 52.6 percent in 2028, and it will be important to monitor for socioeconomic and health care accessibility factors to understand and impact these trends (4). By 2030, WHO's Global Nutrition Target aims for 50 per cent reduction in anaemia prevalence among women of reproductive age (15–49) (5). These figures reflect the country's struggle with poor nutritional standards, iron deficiency, and limited healthcare access. Despite the establishment of iron supplementation programs and fortified food techniques, anemia rates have remained high. For example, iron bioavailability in the Indian diet remains low, ranging from 6% to 12% depending on the demographic category, with factors such as vitamin B12 and folic acid shortages exacerbating the problem. The high incidence rates reflect not just widespread iron deficiency and poor nutritional consumption, but also a lack of access to necessary healthcare services and information. Despite the country's remarkable economic growth in recent decades and the numerous public health initiatives it has implemented to combat the issue, anemia persists in the country today.

National Family Health Survey (NFHS):

The NFHS series is a nationally representative cross-sectional survey conducted by the Ministry of Health and Family Welfare (MoHFW). The survey is the Indian version of the Demographic Health Survey (DHS). The main objective of this survey was to develop state and national estimates for fertility, reproductive health, maternal and child health, HIV-related knowledge, infant and child mortality nutrition, family planning services, women's autonomy, domestic violence, etc (6). National Family Health Surveys (NFHS) used capillary blood samples tested with the portable HemoCue Hb 201 + analyzer. This gadget, which is commonly used in field settings due to its portability and ease of use, delivers instant hemoglobin measurements. In a study by Vart et al. (7), caste based inequalities and anaemia in childhood was studied using NFHS 3 data and it was found that children of scheduled caste (SC) communities are more prone to anemia. Another work by Puranik and N (8) used NFHS-5 data to perform a district level spatial analysis of childhood anemia and found that inadequate nutritional supplementation and lack of healthcare facilities at the district level were important factors for childhood anemia. From NFHS-2 to NFHS-5, the prevalence of mild anaemia (10–10.9 g/dl) increased from 23 percent to 29 percent. NFHS-4 (2015–16) shows that 213 districts in India had more than 65 percent of their youngsters anaemic, which increased to 61 percent (434 out of 707) in NFHS-5 (6). In NFHS-3, prevalence of severe anaemia was 2.9%, in NFHS-4, 1.6%, and in NFHS-5, 2.1%. In NFHS-3, the prevalence of moderate anaemia was 40.4%, which is reduced to 29.2% in NFHS-4 and then increased to 36.7% in NFHS-5. Overall, there was only a slight decrease in the prevalence of severe and moderate anaemia among children over a period of 15 years (from the NFHS-3 to the NFHS-5). Young children aged 12–23 months had the highest prevalence of severe anaemia across the three rounds of NFHS, specifically in NFHS 3 (4.5%), NFHS 4 (2.6%) and NFHS 5 (3.5%) (9).

The National Family Health Survey (NFHS 6) scheduled to begin from July 6 has been dropped from anemia after several experts pointed out that the method used to estimate anaemia in the survey was faulty and led to overestimation. The nfhs estimation problem was that it used capillary samples or blood drawn from a finger prick and measured with an instrument whose results could vary by almost a gram per decilitre of blood. Emerging approaches, such as the Diet and Biomarker Study in India (DABS-I), attempt to increase the accuracy of anemia prevalence statistics by employing venous blood samples and modern analyzers standardized in laboratory settings (10). DABS-I, unlike NFHS surveys which rely on portable devices to estimate hemoglobin levels, uses venous sampling to ensure that inaccuracies of capillary blood testing are avoided. DABS-I survey will map the diet, nutrition and health status and estimate anemia among urban and rural populations using venous samples and autoanalyserd which have been standardized in the ICMR-NIN labs. Although venous sampling presents logistical problems such as controlled transit of samples, laboratory infrastructure and delayed availability of results to participants, it is superior to arterial sampling.

## 3 Current measurement methods

The primary indicator of anemia is hemoglobin concentration, defined by the World Health Organization for age, gender and pregnancy. The diagnosis of anemia is made by comparing hemoglobin (Hb) level, which can be measured in venous or capillary blood, against universally accepted thresholds. In some contexts, either source of blood may be used to estimate population level anemia prevalence or to screen for anemia at the individual level. The gold standard for anemia diagnosis has been established using venous blood assessed by automated hematology analyzer (11). The single drop method is more commonly used in real world Hb measurement settings than pooled capillary blood due to its relative speed of implementation and it requires less materials (microtubes, pipettes, parafilm, etc.). However, there are many sources of variation in Hb estimation that are not all of which can be addressed through good data collection and processing and good quality control procedures in research and surveys (12). When measurement and instrument error and variability are controlled, the estimated mean in Hb concentration of capillary samples is not the same as that estimated from venous samples. This difference is probably due to true biological variability, the magnitude of which is probably dependent on age and sex of participants (11). Capillary blood collection is generally favored by both patients and lab technicians because it is simple, causes minimal discomfort, and is efficient. Results are mostly congruent between capillary blood and plasma, however, it is important to correlate the readings to the respective collection method.

### 3.1 Advantages of capillary blood collection

1. Minimal Blood Requirement: It lowers the risks of complications associated with significant blood loss, particularly in vulnerable patients. For example, ICU patients that have to undergo venous blood sampling may lose up to 2% of their total blood volume per day.
2. Ease of Collection: The finger stick capillary blood collection procedure is less invasive than traditional venous blood draws and is almost painless. Traditional blood draws can be difficult and frustrating with frequent arm veins in children and the elderly.
3. Home Collection: Capillary blood collections at home can be performed by individuals with adequate training using a finger stick method. It is a method that is used by many diabetics to do routine blood sugar checks and is becoming more common among the research and healthcare industries.
4. Varied Collection Sites: Scarring and discomfort can be minimized by using different fingertips or other sampling sites such as the heel of the hand or the upper arm.
5. Growing in Popularity: Capillary blood collection methods using microsampling devices are being recognized and implemented by more labs and research facilities.

### 3.2 Disadvantages of capillary blood collection

1. Test Limitations: Not all laboratory tests can be done on capillary samples.
2. Risk of Rupturing Cells: This may lead to incorrect results due to risk of Rupturing Cells.
3. Potential Complications: Blood sample collection event is associated with both bleeding and infection risks, regardless of the method.
4. Light-headedness: Some patients may be dizzy or faint after the procedure.
5. Scarring: Scarring can occur when the same collection area is repeatedly lanced.
6. Calcified Nodules: These can develop at the collection site especially in infants with repeated sampling. Fortunately, they usually blow away on their own.

### 3.3 Advantages of venous blood sample

1. Accurate Test Results: The preferred method for tests that require larger volumes of blood or require analytes that are present in higher concentrations is venous blood as compared to capillary blood is venous blood draw, as it yields more accurate results.
2. Ability to perform complex tests: Capillary blood collection is often not able to perform a wide range of tests, but venous blood draw is able to perform a wide range of tests, including those that require specialized equipment or techniques that are not possible with Capillary Blood Collection.
3. Lower risk of contamination: The chance of contamination of the sample from external contaminants is less than with Capillary Blood Collection, as the blood is less easily exposed to outside contaminants from the venous blood draw.

### 3.4 Disadvantages of venous blood draw

1. More invasive: Capillary Blood Collection is less invasive than Venous Blood Draw as it does not require inserting a needle into a vein and therefore may not be as painful or cause as much discomfort to some patients.
2. Requires skilled personnel and equipment: A phlebotomist or other healthcare provider trained in collecting blood samples properly, and using sterile equipment to avoid infection or other complications, is needed for venous blood draw.
3. Risk of complications: Venous blood draw has a higher potential to result in a complication like hematoma, nerve damage, or infection, especially if proper technique is not used or when a patient has underlying health conditions rendering him more susceptible to undefined complications.
4. The method is said to be accurate, but there is cost for transportation and processing, delay in reporting of the results, generation of biomedical waste, and discomfort and loss of blood to the patients.

Depending on the specific needs of the patient and the healthcare provider, Capillary Blood Collection and venous blood draw have their own advantages and disadvantages. In order to avoid misinterpretation of Test Results, healthcare professionals need to be aware of when to use each method and how to properly collect blood samples for accurate testing. Healthcare Providers weigh out the pros and cons of each method and choose the best option for their patient and also provide quality care (13).

## 4 Non-invasive methods

Anemia diagnosis requires a complete blood count (CBC) performed through a blood test conducted with a hematology analyzer to determine Hgb levels in venous blood. The development of technological testing methods allows blood examination without requiring any blood removal from the body. The establishment of various non-invasive techniques and devices built with non-invasive technologies promotes understanding of future advances in Non-invasive research. The production of a cost-effective disposable point-of-care (POC) anemia diagnostic device that unskilled people can use for self-testing will empower patients with chronic anemia to check their condition while improving their quality of life and leading to better clinical results and enabling cost-effective anemia screening for the public (14). The WHO issued new guidelines through their March 2024 announcement which recommended laboratory facilities to use automated hematology analyzers and venous blood samples for anemia prevalence estimation (15). Different investigation methods including Pulse co-oximetry and retinal imaging technique and occlusion spectroscopy provide research convenience in this technology study. In this field Masimo Corporation and Orsense Ltd have established themselves as leaders through their developed devices. The technologies from Masimo and Orsense achieve both reduced human staff requirements and enhanced capability to determine essential values. All techniques face difficulties in developing noninvasive procedures that can properly replace invasive ones.Some of the recent digital and non-invasive methods are:

(i) HemoCue: HemoCue devices have been routinely employed to screen for anemia in procedures and clinics as point-of-care devices. It surpasses the copper sulfate method as a donor Hb screening technique. While some users of this technique have indicated that it meets their needs, others have encountered difficulties with HemoCue causing a large number of inappropriate donor rejections. HemoCue utilizes venous and capillary blood to estimate Hb. While this device is effective for large-sample surveys, there are several comparisons with laboratory studies involving venous blood samples that suggest the device is flawed (16).
(ii) The HemoCue® hemoglobin photometer is recognized as a point-of-care device used for estimating hemoglobin concentration in mobile blood donation and critical care areas in health facilities. It is one of the widely used photometers. The Sysmex KX21N is designed as an automated blood cell counter for *in vitro* diagnostics in clinical laboratories. Compact, fully automatic hematology analyzers of this type accomplish simultaneous analyses of 18 parameters in whole blood and capillary blood modes, using a non-cyanide hemoglobin method to determine hemoglobin concentration (17).
(iii) Color-based POC anemia test distinguishes degrees of anemia and provides results through a visual readout snapshot. The device is inexpensive, self-contained, and disposes of the need for additional power or equipment. It offers easy, rapid screening by requiring only a fingerstick of capillary blood (14).
(iv) Considering the global prevalence of anemia, affecting nearly two billion people, especially young children, the elderly, and pregnant women, an innovative smartphone photograph-operated technology serves as a non-invasive screening tool for vulnerable populations, implying major advances in healthcare access. The capacity to sensitively test for anemia without external smartphone attachments or calibration devices dramatically enhances current POC screens for greater accessibility (18).
(v) Masimo Pronto-7 Multiple Wavelength Device: The Pronto-7 developed by Masimo Corporation claims to be more accurate in HB measurement than other Masimo devices. This works by monitoring the pulsatile motion of arterial blood through a finger light probe.
(vi) Spectroscopy of occlusion is one such technique used for measurement of haemoglobin and components of blood which requires non-invasive procedure. The values in question may be established through the signal to noise ratio and light absorption in blood. It has a form of a finger probe with a ring-shaped cuff through which pressure to the finger is applied. Blood flow is occluded momentarily at that position. Signals from multi wavelength light passing through the finger and blood are collected. The range of wavelengths selected is from 600 to 1,500 nm.
(vii) EzeCheck is a portable device which does not require any invasive procedure to measure the concentration of hemoglobin in the body and uses spectrophotometry technique to do so. The sensor is placed on the pulp of the left-hand ring finger of the patient. The device utilizes a cool white light Emitting Diode (LED) to irradiate the finger and collects with EzeCheck, the reflection of the LED light that is reflected from the anterior surface of the finger. These signals are then forwarded to the mobile application. The application connects to a server that runs an AI algorithm to analyze the biomarkers present in the light signal and make predictions on Hgb levels. Then the measurements are sent back to the android application for display to the user.
(viii) Hematology Analyzer (Sysmex XN-1000): Hematology Analyzer is an automatic multi-parameter blood cell counter used for *in vitro* diagnostics in clinical laboratories. It is a compact, fully automated hematology analyzer for multi-parameter blood and capillary blood analysis and has additional features like integrated quality control. This device applies a cyanide-free sodium lauryl sulphate (SLS) method for hemoglobin detection. Results are displayed on the Information Processing Unit (IPU) screen.
(ix) Sahli's Method: Estimation of Hemoglobin using Sahli's technique is quite painless and is an ancient technique. It is a comparator incorporating a brown glass standard which Sahli's graduated percent and gram scaled hemoglobin tube. In this way, blood is combined with an acidic solution and the hemoglobin is transformed into brown-coloured acid hematin. Subsequently, the acid hematin is diluted with distilled water until the color of the acid hematin is identical to the brown-glass standard. The acid hematin value is subsequently determined on the scale.

Not Enough Information on Diagnostic Methods:

## 5 Policy options and implications

### 5.1 Standardizing diagnostic methodologies

#### 5.1.1 Current issues

The variation in hemoglobin measurement methods—such as differences in sample type (venous vs. capillary) and diagnostic devices lead to inconsistent anemia data, hindering effective monitoring and comparison across regions. The absence of standard procedures affects the accuracy of anemia prevalence estimates (11).

#### 5.1.2 Proposed solution

Develop national recommendations for anemia diagnosis in health surveys and clinical settings. Key steps include:

(i) Implementing universal standards for hemoglobin (Hb) testing and blood sample collection, with capillary or venous samples as the standard across field and laboratory settings.
(ii) Adopting dependable diagnostic procedures, such as the cyanmethemoglobin method, for consistency in field investigations and clinical laboratories.
(iii) Regularly calibrate and standardize diagnostic equipment to assure accuracy across facilities.

#### 5.1.3 Implications

Standardizing diagnostic techniques will increase the reliability and uniformity of anemia prevalence statistics, enabling improved evaluation of health measures and progress monitoring. This consistency would also allow scholars and politicians to effectively evaluate intervention success rates, laying the groundwork for evidence-based decision

### 5.2 Improving Non-invasive diagnostic devices

#### 5.2.1 Current issues

Non-invasive anemia diagnoses, such as mobile applications and Masimo Pronto, are gaining popularity, particularly in field settings. However, studies reveal that these devices have a weaker correlation with reference to hemoglobin values than classic invasive methods such as HemoCue analyzers, which could contribute to mistakes in prevalence estimations (19).

#### 5.2.2 Proposed solution

Allocate funding for the research and development of non-invasive anemia diagnostics to improve accuracy and reliability. Specific focus areas could include:

(i) Increasing accuracy to levels comparable with invasive methods while maintaining the simplicity and affordability of non-invasive devices.
(ii) Collaborating with technology developers to improve device algorithms, ensuring they account for variables such as skin tone and environmental factors that may impact readings.
(iii) Conducting trials in diverse field settings to validate device performance across different demographics and geographical conditions.

#### 5.2.3 Implications

Large-scale, precise anemia screening would be made easier in rural and isolated locations where conventional laboratory equipment is frequently unavailable using enhanced non-invasive devices (20). Additionally, health professionals may direct treatments and interventions to high-prevalence areas without requiring laboratory support with the help of high-precision non-invasive diagnostics, which would speed up diagnosis and improve resource allocation.

### 5.3 Enhancing accessibility in resource-limited settings

#### 5.3.1 Current issues

High-quality anemia diagnostics, such as automated hematology analyzers, are frequently unavailable in rural and low-income communities where anemia is most prevalent. Diagnostic options are limited due to resource and infrastructure constraints, such as a lack of skilled workers and access to laboratory facilities.

#### 5.3.2 Proposed solution

Implement targeted programs to improve access to anemia diagnostics in rural and underserved areas, including:

(i) Subsidizing equipment such as portable HemoCue analyzers to make them affordable for rural health centers.
(ii) Training healthcare workers on the use of both non-invasive and minimally invasive devices, equipping them with the knowledge to operate these tools accurately and efficiently.
(iii) Providing government funding to supply diagnostic tools for remote villages, improving access where fixed health facilities are scarce.

#### 5.3.3 Implications

Increased access to anemia diagnostics would allow for extensive screening and early treatments, especially in high-prevalence areas. This will also aid rural areas in detecting anemia-related issues earlier, minimizing health disparities between urban and rural populations. Affordable and accessible diagnostics result in improved anemia management, potentially lowering anemic prevalence in the most affected communities.

## 6 Strengths

The Research year has witnessed a rising interest in developing noninvasive technologies to optimize and simplify hemoglobin screening processes. Multiple medical devices, including digital hemoglobinometers (TrueHb and HemoCue) and noninvasive technologies like Masimo Pulse Oximetry, Orsense NBM 200, and other spectroscopy-based devices, demonstrate potential applications in public health (21). The direct cyanmethemoglobin method stands as the International Council for Standardization in Haematology's gold standard for hemoglobin estimation in clinical laboratories and represents the most successful and cost-effective approach, and when combined with the WHO's revised protocol and portable device calibration, it can effectively reduce inter-survey variability (22). The recommended procedure for measuring hemoglobin concentration includes venous blood sampling with automated haematology analyzers while using high-quality control practices to monitor results in individuals and populations. Multiple blood drops collected through a single capillary prick might serve as a method to reduce the variability that results from different blood collection methods (23). The TrueHb haemometer test functions as a point-of-care (POCT) device for hemoglobin (Hb) estimation to bridge the diagnostic gap for pregnant women's anemia screening at primary health centers and community settings (24). The procedure for hemoglobin estimation needs minimal training for staff members. The primary care level diagnosis of cases will reduce the workload at secondary and tertiary healthcare facilities (22). A study at AIIMS New Delhi, combined with IIPH-Delhi, PHFI, and MoHFW demonstrated that TrueHb haemometer yielded better diagnostic results than HemoCue Hb 301 system and non-invasive devices for field-based patient anemia screening (25). The adoption of noninvasive techniques reduces the requirement for finger pricks while getting rid of biomedical waste, therefore establishing the technology as an essential hemoglobin assessment tool. The Pronto-7 pulse CO-oximetry device (Masimo Corporation) used in preoperative assessment revealed noninvasive technologies as important because they allow quick anemia detection and treatment at the initial clinical visit (26).

## 7 Limitations

Despite the strengths, Digital haemoglobinometers currently face limitations because their screening capabilities are restricted to specific groups of beneficiaries, but states are working on plans to extend the application to other beneficiaries. Digital haemoglobinometers remain confined to resource-constrained settings because they have limited availability and their recurring consumables, including strips, cuvettes, batteries and lancets for pricking, are expensive (21). The dread of receiving needle pricks among adolescents creates another barrier that prevents them from taking part in screening procedures. The differences in hemoglobin measurements between capillary and venous blood tests exist, but the exact extent of this variation remains unknown. The conversion of hemoglobin concentration from capillary blood to venous blood samples remains impossible because of the lack of data (23). Digital haemoglobinometers are widely available across the market, with multiple brands accessible through the Government e-Marketplace portal. The process of ensuring high quality with specific and sensitive performance in these devices proves difficult to achieve. Research conducted using indirect methods produced conflicting results that either increased or decreased the prevalence estimates of anemia (27).

Non-invasive hemoglobin measurement requires a cautious interpretation, especially in patients like those in shock or under vasopressor therapy, who have poor peripheral perfusion. These states can lead to inaccurate readings. Furthermore, devices that measure hemoglobin non-invasively need their subjects to be very still during the measurement process. Additionally, there are many devices in existence for testing anemia that are accessible at the point of care, however, most of these systems are confined to a laboratory or a doctor's office because they are so expensive and because they demand a technician with a certain level of expertise (14). The direct cyanmethemoglobin method represents a gold standard, but its operation demands spectrophotometer technology that exists mainly in laboratory facilities. A staff member needs specialized training with strict methodology guidelines for performing the indirect cyanmethemoglobin method because this method remains beneficial when laboratory facilities are distant from collection sites and no laboratory setup exists (22). The promising non-invasive devices EzeCheck and NiADA need larger multi-center trials, which must include diverse skin tones and ages in various environments. The use of hematology analyzers demands a medical setup for operation and takes three to four hours for result processing, while the entire process becomes expensive because of reagents, technician salaries, and machine maintenance costs (21).

## 8 Cost-effectiveness

Cost-effectiveness acts as an essential measure during device assessment processes. Healthcare facilities make their treatment decisions heavily influenced by the expenses needed to acquire diagnostic equipment alongside operation and maintenance costs (21). The establishment of infection-related hemoglobin concentration alterations requires cost-effectiveness data to determine practicality (23). EzeCheck training expenses, along with support costs, remain substantially lower than those of Masimo Rad-67, which proves its unsuitability for limited-resource healthcare environments due to its higher operational costs. The cost analysis included factors related to test execution convenience, invasive or noninvasive testing methods, consumable usage, charging requirements, and the total device price. Noninvasive devices offer better affordability and operational accessibility when compared to invasive devices because they lead to zero patient discomfort while discarding waste at a minimal setup level and delivering swift test outcomes for prompt disease identification (21). The TrueHb device presents an initial low price, while its continuous operation costs surpass those of HemoCue. The use of the cyanmethemoglobin method proves more cost-effective than the TrueHb device because the stripe used in the TrueHb device is expensive compared to the price of filter paper, tips, and Drabkin's Reagent. Khurana et al. (22) found that the filter paper method represents a cheaper option for detecting anemia.

## 9 Stakeholder views on policy options

Stakeholders must consider both blood source and analyzer type when measuring hemoglobin levels and tracking changes in anemia rates between periods or different settings, especially when venous blood or automated analyzers are unavailable (23). The Government of India launched Anemia Mukt Bharat (AMB) as a national initiative to establish systematic approaches for anemia prevention, detection, and management strategies (28). “AnemiaPhone” technology, developed by Cornell researchers, was transferred to the Indian Council of Medical Research for national integration into anemia prevention and women's health and maternal child health programs across India (29–34). The “Anemia Mukt Bharat” program has implemented digital meters for routine screening of school-going adolescents and pregnant women to achieve major advancements in proactive health care. These initiatives enable timely anemia detection and promote knowledge about anemia status across different stakeholder groups, including healthcare workers and policymakers. Through its evidence-based interventions and expert knowledge, PATH effectively contributes to decreasing the anemia burden. It helps policymakers and manufacturers develop appropriate point-of-care devices for anemia screening through diagnostic evaluation processes. PATH led assessments in 15 states with The Inclen Trust International and UNICEF and the Institute of Economic Growth to evaluate the AMB program's enablers and barriers, and facilitators. PATH's commitment to health care innovation through user-driven enhancements is reflected in the collaborative work that resulted in operational research for non-invasive Hb meters designed for anemia screening (28).

## 10 Requirements & barriers to implementation

### 10.1 Target group

Socioeconomic disadvantages are concentrated in urban areas. Even though income poverty is an urban phenomenon, many urban dwellers are poor in access to basic services. The anxiety caused by needle sticks among adolescents' functions as a major barrier that prevents them from engaging with screening activities. To address this, non-invasive screening methods based on scientific evidence should be developed to enhance the participation rate of this demographic. An accurate anemia diagnosis must remain accessible and available for patients whenever their need for diagnosis arises, without consideration of their geographical location, cultural background, or economic status (23). The ability of women and adolescent girls to access health services and obtain enough nutritious food is limited by both economic barriers and household cultural norms that influence their decision-making. The accessibility of hemoglobin testing depends on three key factors: geographical location of testing facilities, price of testing services, and culturally appropriate communication approaches. The development of culturally suitable communication strategies remains essential to provide accurate evidence-based information about anemia diagnosis and its significance for health development to avoid any misconceptions. National and subnational programs must align their approaches to specific cultural characteristics of their target populations because this strategy increases program adoption and sustainability. Health programs achieve better acceptability and adoption rates through simple information systems that provide easy access to content understandable across different population groups. The method of information delivery should be designed to create a perception of normative statements as suitable for every stakeholder involved.

### 10.2 Providers

Although the indirect cyanmethemoglobin method provides a simple implementation for sample testing, it requires trained staff to follow strict methodology because laboratory equipment might be distant from sample collection points (22). The lack of proper training regarding hemoglobinometer operation leads to decreased use of these devices. Additionally, the ground-level troubleshooting systems require improvements, and access to biomedical maintenance and recalibration services are insufficient (23). The process of hemoglobin estimation demands minimal training for the staff members involved.

### 10.3 Managers

Device shortages restrict screening programs from reaching their full capacity because they limit the number of individuals who can undergo testing. The lack of test strips disrupts screening activities because it causes shortages of screening consumables, which reduces program effectiveness (28). Screening reports that fail to link with appropriate treatment interventions result in untreated anemia cases, thus demonstrating the essential importance of integrated referral systems to provide proper care for patients identified with anemia. Multiple screening devices with inadequate quality assurance systems produce unreliable results because their accuracy levels differ between devices (23). The direct diagnostic method faces implementation challenges due to venous blood sample collection requirements, storage, and transportation needs to laboratory facilities, which restrict its use in constrained PHC facilities and field settings and large survey operations (22).

### 10.4 Policymakers

The selection of iron deficiency anemia interventions, including supplementation, fortification, nutrition education, and their combined approaches, requires policymakers, program implementers, and clinicians to evaluate micronutrient status along with dietary variations and cultural practices and food processing methods, and economic factors at regional and local levels. Special attention needs to be given to the potential negative outcomes from receiving too much iron because such situations commonly appear in malaria-endemic areas or communities with haemoglobinopathies. Knowledge of the underlying causes of anemia enables us to develop appropriate intervention strategies for treatment, along with prevention and reduction of this condition. Policymakers aiming to decrease anemia prevalence should recognize local conditions and deploy various suitable interventions (such as iron and malaria prevention strategies) to address these multi-faceted factors. A complete intervention approach will help achieve targeted results while preventing unwanted side effects and maintaining acceptable iron consumption levels across all dietary sources. The evaluation of iron intervention results requires complete information about biomarkers of iron status, including ferritin and serum transferring receptor levels and program execution data, and signs of inflammation. It is advisable to examine additional biomarkers that would help identify the root causes of anaemia. The accessibility and availability of precise diagnostic tools should receive promotion whenever identified needs arise without consideration for economic or cultural situations or geographical characteristics (23).

## 11 Actionable recommendations

### 11.1 Enforce uniform standards for hemoglobin testing

Develop and enforce national guidelines requiring the use of standardized techniques for hemoglobin measurement in health surveys and clinical settings. Standardization should include capillary and venous sample protocols and calibration and quality control criteria.

### 11.2 Funding for non-invasive device development

Invest in research to improve the precision of non-invasive diagnostics, making them more useful in field settings where invasive procedures are impractical. Encourage collaborations with technology developers to improve device algorithms for greater accuracy across diverse populations and environments.

### 11.3 Expand access to portable diagnostic tools in rural areas

Implement government-funded programs to deliver diagnostic instruments such as HemoCue analyzers in low-income healthcare facilities. Additionally, provide mobile diagnostic devices to provide diagnostic coverage to outlying locations with no set healthcare infrastructure.

### 11.4 Develop educational campaigns for anemia prevention and screening

Launch public health campaigns to raise awareness and prevent anemia. Campaigns can educate communities on nutritional practices for preventing anemia and emphasize the necessity of regular screening, promoting early detection and treatment.

### 11.5 Strengthen data collection and reporting for anemia surveillance

Integrate anemic data gathering into current health monitoring frameworks to ensure consistent reporting across sites. Encourage venous and capillary hemoglobin data collection in national surveys to improve accuracy and fill gaps when resources are restricted. Accurate data reporting will allow for rapid policy modifications, increasing the responsiveness of public health initiatives.

## 12 Conclusions

Anemia remains a major public health concern in India, with severe implications for health and productivity, especially among women and children. Enhancing the current situation of anemia diagnostics through standardized practices, technological advancement, and expanded accessibility is crucial for effectively addressing this issue. Standardized diagnostic techniques will ensure that statistics on the incidence of anemia are reliable and comparable, allowing for more effective policymaking and resource allocation. Improvements in non-invasive equipment will improve diagnostic capabilities in locations with limited standard laboratory infrastructure. Finally, offering accessible diagnostic instruments in impoverished areas will allow local healthcare providers to diagnose anemia earlier, increasing treatment outcomes and contributing to the country's overall decline in anemia rates. By implementing these policy suggestions, India can make substantial strides toward lowering anemia rates and guaranteeing equitable access to quality healthcare for all citizens.
